# Morphological Diversity of the Rod Spherule: A Study of Serially Reconstructed Electron Micrographs

**DOI:** 10.1371/journal.pone.0150024

**Published:** 2016-03-01

**Authors:** Shuai Li, Joe Mitchell, Deidrie J. Briggs, Jaime K. Young, Samuel S. Long, Peter G. Fuerst

**Affiliations:** 1 University of Idaho, Department of Biological Sciences, Moscow, Idaho, 83844, United States of America; 2 North Idaho College, Natural Sciences Division, Coeur d’Alene, Idaho, 83814, United States of America; 3 Lewis-Clark State College, Department of Computer Sciences, Lewiston, Idaho, 83501, United States of America; 4 WWAMI Medical Education Program, Moscow, Idaho, 83844, United States of America; Dalhousie University, CANADA

## Abstract

**Purpose:**

Rod spherules are the site of the first synaptic contact in the retina’s rod pathway, linking rods to horizontal and bipolar cells. Rod spherules have been described and characterized through electron micrograph (EM) and other studies, but their morphological diversity related to retinal circuitry and their intracellular structures have not been quantified. Most rod spherules are connected to their soma by an axon, but spherules of rods on the surface of the *Mus musculus* outer plexiform layer often lack an axon and have a spherule structure that is morphologically distinct from rod spherules connected to their soma by an axon. Retraction of the rod axon and spherule is often observed in disease processes and aging, and the retracted rod spherule superficially resembles rod spherules lacking an axon. We hypothesized that retracted spherules take on an axonless spherule morphology, which may be easier to maintain in a diseased state. To test our hypothesis, we quantified the spatial organization and subcellular structures of rod spherules with and without axons. We then compared them to the retracted spherules in a disease model, mice that overexpress *Dscam* (Down syndrome cell adhesion molecule), to gain a better understanding of the rod synapse in health and disease.

**Methods:**

We reconstructed serial EM images of wild type and *Dscam*^*GoF*^ (gain of function) rod spherules at a resolution of 7 nm in the X-Y axis and 60 nm in the Z axis. Rod spherules with and without axons, and retracted spherules in the *Dscam*^*GoF*^ retina, were reconstructed. The rod spherule intracellular organelles, the invaginating dendrites of rod bipolar cells and horizontal cell axon tips were also reconstructed for statistical analysis.

**Results:**

Stereotypical rod (R1) spherules occupy the outer two-thirds of the outer plexiform layer (OPL), where they present as spherical terminals with large mitochondria. This spherule group is highly uniform and composed more than 90% of the rod spherule population. Rod spherules lacking an axon (R2) were also described and characterized. This rod spherule group consists of a specific spatial organization that is strictly located at the apical OPL-facing layer of the Outer Nuclear Layer (ONL). The R2 spherule displays a large bowl-shaped synaptic terminal that hugs the rod soma. Retracted spherules in the *Dscam*^*GoF*^ retina were also reconstructed to test if they are structurally similar to R2 spherules. The misplaced rod spherules in *Dscam*^*GoF*^ have a gross morphology that is similar to R2 spherules but have significant disruption in internal synapse organization.

**Conclusion:**

We described a morphological diversity within *Mus musculus* rod spherules. This diversity is correlated with rod location in the ONL and contributes to the intracellular differences within spherules. Analysis of the *Dscam*^*GoF*^ retina indicated that their R2 spherules are not significantly different than wild type R2 spherules, but that their retracted rod spherules have abnormal synaptic organization.

## Introduction

Phototransduction initiates with the absorbance of photons in retinal rods, cones and ganglion cells. Most species have a binary retina, with visual stimuli largely transmitted from the rod and cone pathways, which then send signals to downstream neurons. The synaptic terminal of the rod photoreceptor plays a crucial role in the initiation of scotopic visual signals and can also signal through an alternative pathway when directly coupled to cone terminal telodendria through gap junctions [1–3]. Rod cells form ribbon synapses with the tips of horizontal cell (HC) axons and the dendrite tips of rod bipolar cells (BPCs) in a wide range of mammalian species, which invaginate into the rod spherule [4–9].

While rod spherules are remarkably uniform compared to cone synapses [9], several prominent differences between rod spherules within individuals of several species have been reported. A small number of rods are occasionally contacted by the dendrites of cone bipolar cells, including bipolar cell types 3a, 3b and 4 in the mouse, providing a cone independent pathway through which the rod visual pathway can interact with the cone driven photopic visual pathway [10–13]. Morphological variation between the rod spherules of a given species have also been noted within the rabbit retina, specifically if the spherule is connected to the soma through an axon, and within several species of mice in the genus *Sylvaemus* [5, 8].

The stereotypic placement of rod spherules is disrupted in a large number of mouse mutants [14] and acute trauma models, which typically manifests as retraction of the rod axon and formation of synaptic contacts within the outer nuclear layer of the retina. These include models of retinal detachment [15, 16], physiological abnormality [17–24], absence of horizontal cells [25, 26], perturbation of cell adhesion [27–29], lack of ribbon synapse components [30] and in the aging retina [31–33]. Several of these studies were able to confirm the presence of synaptic components adjacent to the rod soma by electron microscopy, and careful analysis in one study of single micrographs led the authors to conclude that synapses located in the outer nuclear layer are similar to those in the outer plexiform layer [34].

In this study, we utilized libraries of electron micrographs (EM) generated by scanning block face electron microscopy to reconstruct detailed 3D structures from both wild type (WT) and *Dscam*^*GoF*^ retinas to characterize rod synapse diversity and test if retracted spherules in the *Dscam*^*GoF*^ retina fell within the normal spectrum of rod spherule diversity. First, we investigated rod spherule diversity in the *Mus musculus* retina, including organelle and synaptic organization. We report structural and organizational differences between two morphological rod spherule types. Typical axon-bearing rod spherules (R1) accounted for most spherules, while a small number of spherules (R2) lacked an axon and hugged the rod cell soma. Second, we found that R2 rod spherule morphology is not significantly different in the *Dscam*^*GoF*^ retina compared to wild type. Finally, we characterized displaced rod spherules in *Dscam*^*GoF*^ mice, those located within the outer nuclear layer. We found that despite appearing morphologically similar to R2 rod spherules, that the retracted spherules have abnormal synaptic organization, indicating that these rods are not simply taking on an R2 type morphology. The results of this study provided new insight of how rods are organized in the outer retina.

## Results

### Stereotypical rod spherules have highly uniform morphology

**Fig 1:**
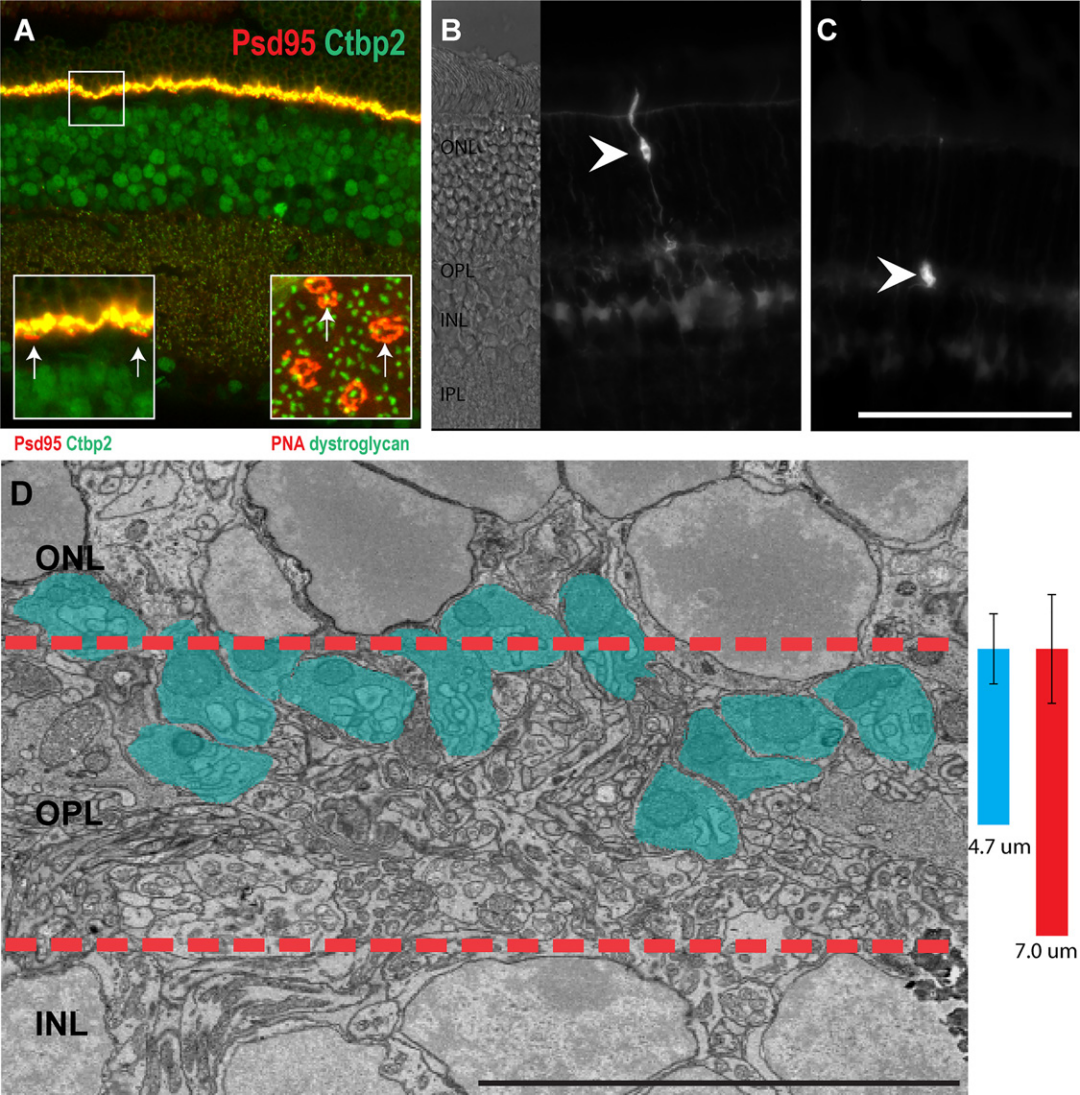
Location of the photoreceptors and their axon terminals. **A**, Retina stained with PSD95 and CTBP2. The left inset in **A** depicts higher resolution image to more clearly visualize the cone terminals (arrows). The right inset, stained with PNA to label cone terminals, and dystroglycan to label rod synapses, depicts the horizontal distribution of rods and cones (arrows). **B**, A single photoreceptor, located in the middle of the ONL, displays a stereotypical morphology with a long axon extending from the cell soma into the outer plexiform layer (OPL). **C**, A single photoreceptor residing on the apical surface of the ONL (arrow head) lacks an axon connecting its synaptic structure and soma. **D**, An electron micrograph of the OPL region (between the red dashed lines). Blue colored areas are rod spherules. The blue bar refers to the depth of projection for rod terminals in the central mouse retina (4.7μm standard deviation = 0.92μm), the red bar refers to the total thickness of the OPL, as measured in the central retina (7μm standard deviation = 1.43 μm. n = 13, at postnatal day 42. Scale bar in **A** is 100 μm in **A**-**C**, 50 μm in zoom in view of **A**, Bar in **D** is 10 μm).

Rods are the dominant photoreceptor in the mouse retina and account for the vast majority of photoreceptors, typically 10–12 cell bodies in thickness [35]. Unlike the inner plexiform layer (IPL), which is functionally subdivided into many layers, the OPL is a relatively thin synaptic layer containing large cone terminals and smaller rod terminals (Fig 1A). Rod and cone photoreceptors have a highly regular morphology in which the cell body is located in the ONL and projects an axon into the OPL, where it synapses with bipolar and horizontal cells (Fig 1B). This morphology represents most rods in the wild type mouse retina, and we classified this group of rod spherules as type 1 (R1). Rods without axons commonly reside at the apical surface of the ONL, and to distinguish these axonless rod spherules, we classified them as type 2 rod spherules (R2) (Fig 1C). Viewed by electron microscopy, the R1 rod terminals are conspicuous due to their large mitochondrium (Fig 1D) and are localized in approximately the top 67% of the OPL (Fig 1D).

**Fig 2:**
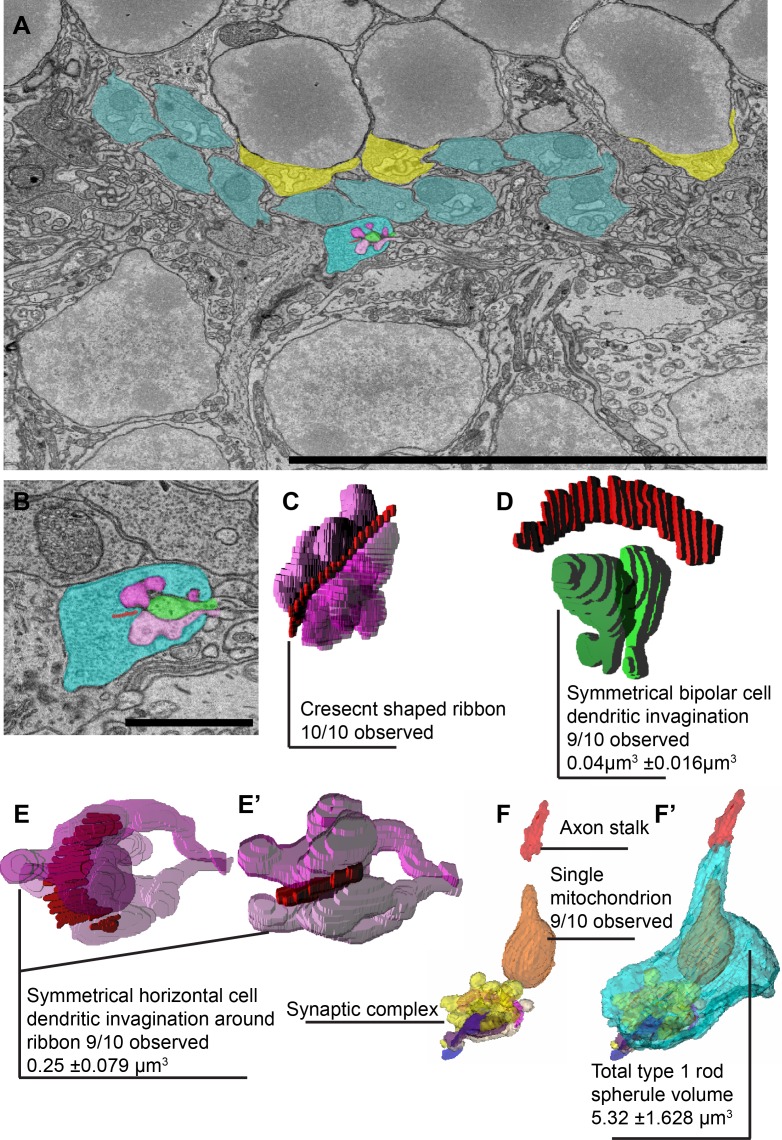
Internal structure of a stereotypical R1 rod spherule. **A**, EM image of WT retina focused on the outer plexiform layer (OPL). R1 rod spherules are colored in blue and R2 rod spherules are colored in yellow. The center spherule is highlighted for reconstruction. **B**, High magnification of the EM image with each intracellular structure colored (red is the ribbon, green is invaginating bipolar cell dendrites, purple is invaginating horizontal cell axons). **C**, 3D reconstruction of R1 ribbon and invaginating horizontal cell axons. **D**, 3D reconstruction of R1 ribbon and invaginating bipolar cell dendrites. Note the symmetry of the bipolar dendrites. **E and E’**, Ribbon with horizontal cell axons from a different angle show the two invaginating axon tips maintain a spatial symmetrical orientation. **F and F’**, R1 rod spherule reconstruction modeled without and with cytoplasm region to show internal structures. (Scale bar in **A** is 10 μm, in **B** is 1.6 μm).

R1 spherules were reconstructed in high-resolution serial EM images to quantify the morphological parameters of the rod spherules and associated structures (Fig 2A). The individual rod spherule was well organized and its substructures were easily identified and reconstructed (Fig 2B). R1 spherules generally contained one crescent-shaped ribbon (Fig 2C, 10/10). Typically, it contacted two bipolar cell dendritic invaginations (9/10) with a size of 0.04 ±0.016 μm^3^ (Fig 2D, S1 Movie, n = 19). Horizontal cell axon tips formed a spatially symmetrical invagination around the bipolar cell dendrites with a size of 0.25 ±0.079 μm^3^ (Fig 2E and 2E’, S2 Movie, 9/10 spherules, n = 19 HC axon tips). This complex synaptic terminal is formed at the distal end of the spherule, located near a single large mitochondrium, averaging 0.84 ±0.17μm^3^ in size (Fig 2F and 2F’, n = 10). The total R1 spherule averaged 5.319 ±0.7μm^3^ in volume from the end of the axonal stalk. The cytoplasm, bipolar cell dendrites, horizontal cell axons and mitochondria composed 73±4%, 0.7±0.3%, 4±1.5% and 16±3% of the total volume respectively (n = 10). The internal organization is highly uniform (S3 Movie).

### R2 rod spherules

**Fig 3:**
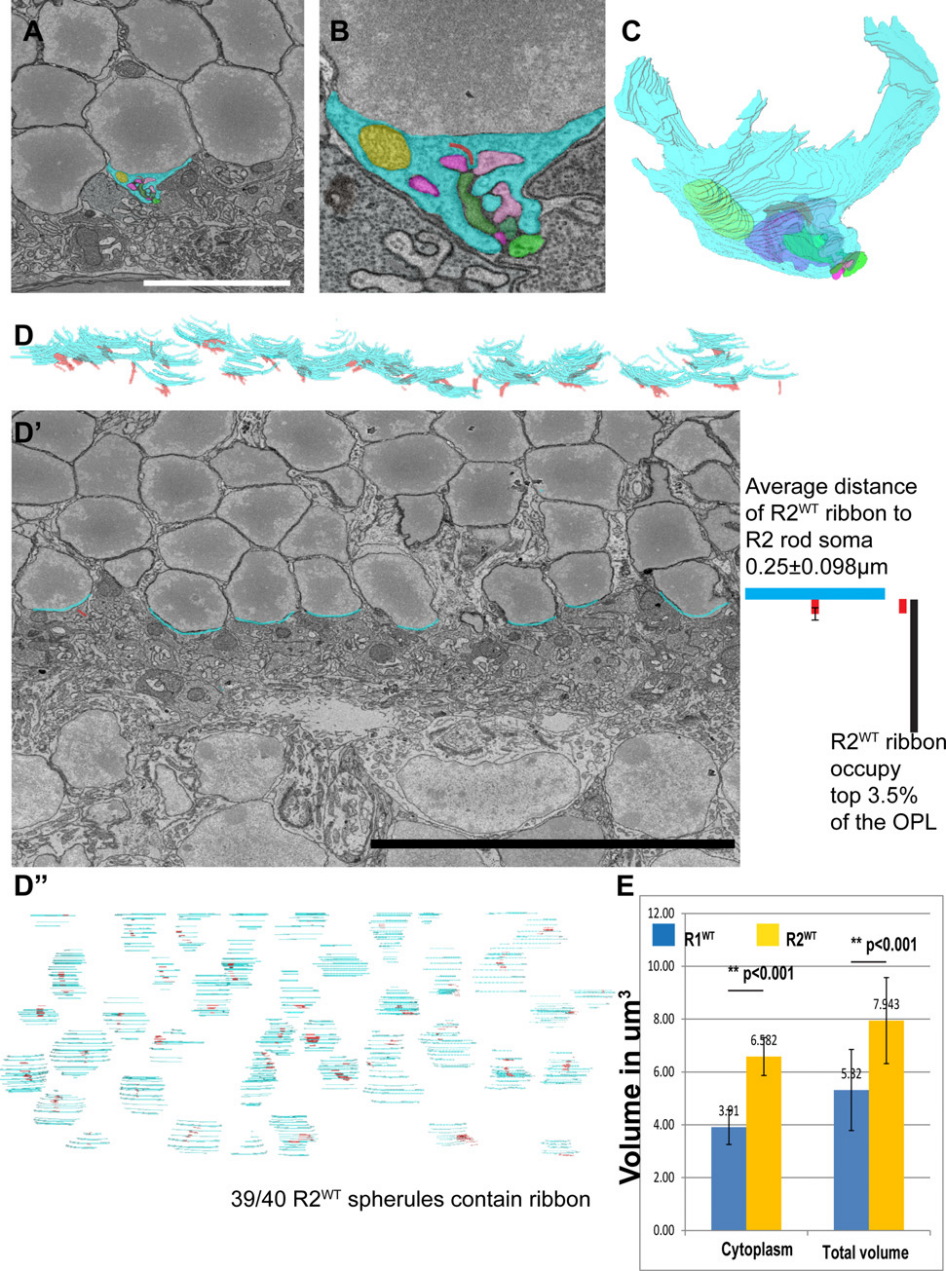
The apical layer of ONL rods primarily have an R2 spherule configuration. **A**, WT type 2 rod spherule (R2) is adjacent to the rod soma. **B**, A high magnification image of the R2 spherule. **C**, 3D reconstruction of the R2 spherule. **D-D”**, Overview of R2 spherules for all rods in a volume. **D**, Side view of 3D reconstruction of the R2 rod surfaces, in which all R2 rods reside at the basal layer of the OPL and do not project axons. **D’**, Single EM image of the bottom surface of type 2 rods. The distance between the R2 nucleus surface and the middle of ribbons was measured. **D”**, En face view of R2 spherule reconstruction with attached ribbons. **E**, Quantification shows type 1 and type 2 rod spherules are significantly different in cytoplasm and total volumetric measurement (n = 10, cytoplasm R1 std dev = 0.6, R2 std dev = 1.5, total volume std dev R1 = 0.7, R2 std dev = 1.6 unit in μm^3^). Scale bar in **A** is 7μm in **A**, 1.7μm in **B**. Scale bar in **D’** is 20μm.

In addition to the stereotypical R1 spherule, we found that rods located on the surface of the ONL and adjacent to the OPL do not project axons. Instead, their synaptic structures were located next to the rod nucleus (Fig 2A and Fig 3A). We classified this group of rod spherules as R2 rod spherules. We identified subcellular structures and observed a distinct morphology in the R2 spherule compared to the R1 spherule. The most obvious difference in the R2 spherule was the lack of an axon and that the spherule was bowl shaped (Fig 3B and 3C and S4 Movie). Whole R2 surfaces with their correlating ribbons were reconstructed (Fig 3D and S5 Movie), and the distance of the ribbon centers to the nucleus was measured. The average distance from the R2 ribbon to the nucleus was 0.25 ±0.098μm. The R2 synapses appeared only on rods located at the apical surface of the OPL layer (Fig 3D’, the distance equivalent to 3.5% of the overall thickness of the OPL). A whole mount view of the R2 surface reconstruction indicated 95% (92/97) of apical rods (those on the INL/OPL boundary) had an R2 spherule (Fig 3D”). Statistical analysis of the cytoplasm volume and the total volume of the R1 and R2 spherules indicated a significant difference comparing the two spherule types (Fig 3E). The R2 morphology was observed in all EM stacks, EM image collections, and in independently generated image stacks (S1 Fig).

### Synaptic organization but not volume is preserved in R2 spherules

**Fig 4:**
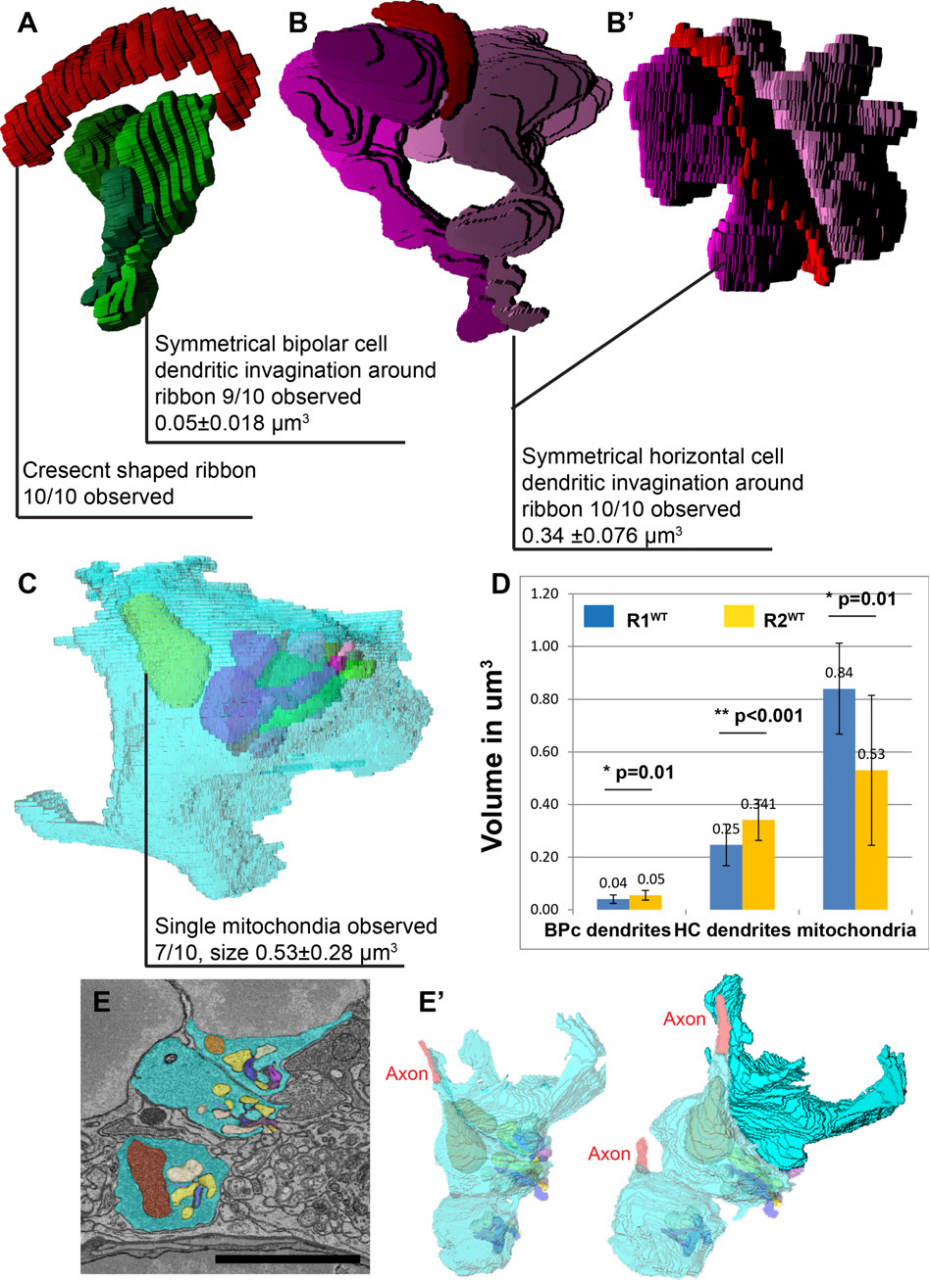
R2 spherules have a similar complement of invaginating neurites but are morphologically distinct from R1 rod spherules. **A**, 3D reconstruction of R2 ribbon contacting invaginating bipolar cell dendrites indicated a similar synaptic symmetry to R1 spherule organization. **B-B’**, R2 ribbon contacting invaginating horizontal cell axon tips shows a similar symmetry to R1 spherule organization. **C**, En face view of a reconstructed R2 spherule. **D**, Statistical analysis indicated the volumetric difference in internal organelles including bipolar cell dendrites, std dev R1 = 0.016 μm^3^, R2 = 0.018 μm^3^, HC axon tips, std dev R1 = 0.079 μm^3^, R2 = 0.077 μm^3^, Mitochondria volume std dev R1 = 0.17 μm^3^ and R2 = 0.29 μm^3^. **E**, R1 and R2 spherules are adjacent to each other. **E’**, 3D reconstruction of the spherules indicated a close spatial organization of R1^WT^ and R2^WT^ spherules. Scale bar in **E** is 3.6 μm.

We reconstructed the internal structures of R2 spherules to determine if synaptic structure was conserved when comparing R1 and R2 spherules. Ribbons in R2 spherules still maintained a well-organized morphology (10/10), as did the invaginating bipolar cell dendrites with a volume of 0.05 ±0.018μm^3^ (Fig 4A, 9/10, n = 21). The invaginating horizontal cell axons followed a similar pattern with a degree of spatial symmetry and a volume of 0.34 ±0.076 μm^3^ per axon tip (Fig 4B and 4B’ 10/10, n = 20). Significantly smaller mitochondria were observed in R2 spherules with a size of 0.53 ±0.28μm^3^ (Fig 4C, 7/10).

The volume of internal structures within the R1 and R2 spherule was significantly different. R2 had larger invaginating neurites from interneurons but smaller mitochondria (Fig 4D, n>10, *t*-test bipolar cell dendrites p = 0.01, horizontal cell dendrites p<0.001, mitochondria p = 0.01). However, the actual composition and spatial organization of the internal structures were highly similar between the two types of rod spherules. The total R2 spherule volume was 7.94 ±1.63 μm^3^. The cytoplasm, bipolar cell dendrites, horizontal cell axons and mitochondria composed 82±4%, 0.7±0.02%, 4±0.8% and 7±4% of the total volume respectively (n = 10) (Fig 4E and 4E’ and S6 Movie).

### The *Dscam*^*GoF*^ retina has R1, R2 and retracted spherules

**Fig 5:**
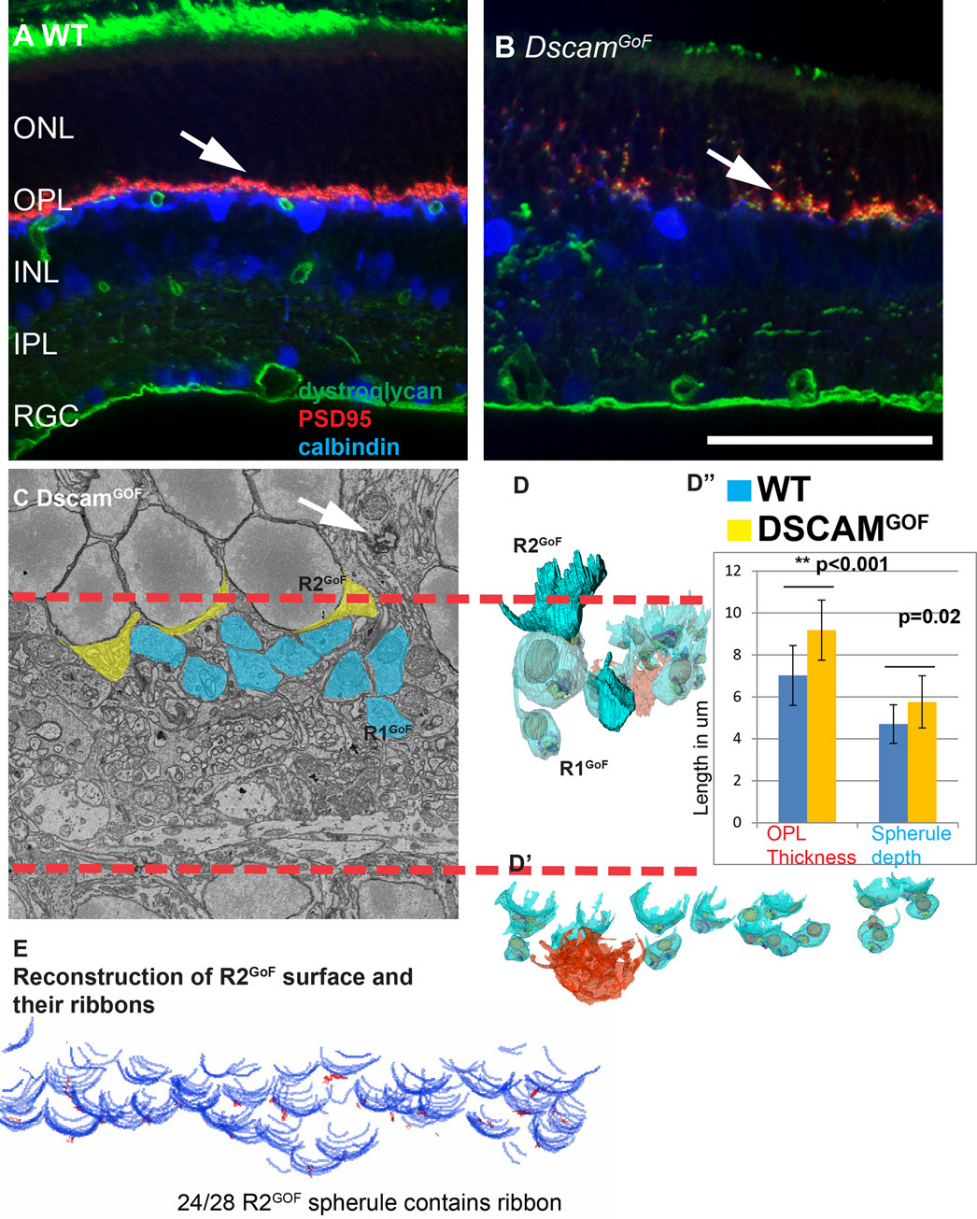
*Dscam*^*GoF*^ retina contains R1 and R2 spherules with morphologies typical of these spherule types. **A**, WT retina stained with dystroglycan, PSD95 and calbindin. HC axon tips are projected evenly in the OPL and no synaptic markers are observed within the ONL (arrow). **B**, *Dscam*^*GoF*^ retina showing misplaced synaptic markers as well as the outgrowth of the horizontal cell axon tips (arrow). **C**, Colored electron micrograph showing R1 and R2 spherules in the *Dscam*^*GoF*^ retina. **D-D’**, Reconstructed R1 spherules in the *Dscam*^*GoF*^ retina with a reconstructed cone pedicle (in orange). **D”**, *Dscam*^*GoF*^ OPL parameters. **E**, R2 rod basal surface reconstruction in the *Dscam*^*GoF*^ retina. Most rods on the surface of the *Dscam*^*GoF*^ retina have an R2 spherule morphology. The scale bar in B is equivalent to 100 μm in **A** and **B** and 10 μm in **C**.

To understand synaptic abnormality related with retinal diseases and photoreceptor plasticity, we used the *Dscam*^*GoF*^ transgenic mouse as a model. This mouse strain has misplaced synapses located within the ONL that retract over a similar time course as those observed in other models of rod retraction [27]. This results in the presence of retracted rod spherules located within the ONL (Fig 5A and 5B).

We measured the parameters of the *Dscam*^*GoF*^ OPL and identified R1 and R2 rod spherules, classified as R1^GoF^ and R2^GoF^ spherules. R2^GoF^ spherules were consistently adjacent to the ONL with no axons, and R1^GoF^ spherules were consistently closer to the inner retina compared to the R2^GoF^ spherules, suggesting a consistency with the WT morphology (Fig 5C, 5D and 5D’ and S7 Movie). The OPL layer in *Dscam*^*GoF*^ is 30% thicker and spherules project 20% deeper than WT controls (Fig 5D”, 9.1 vs 7.0 μm, p = 0.0004, n = 13. And 5.7 vs 4.7 μm n = 13: Student’s *t*-test p = 0.02). Rods on the surface of the ONL/OPL boundary mostly had R2 synapse organization (24/28) (Fig 5E).

**Fig 6:**
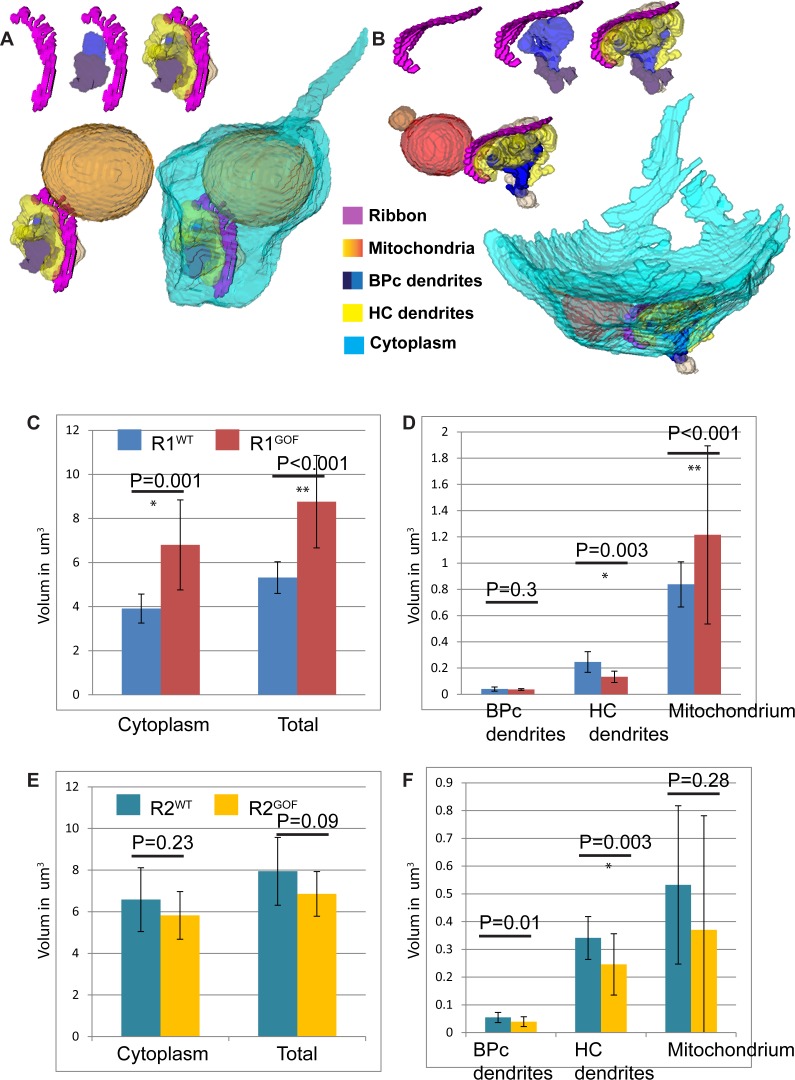
R1 spherules in *Dscam*^*GoF*^ retina are significantly different from R1 spherules in WT, while R2 spherules in *Dscam*^*GoF*^ do not differ significantly from their WT counterparts. **A and B**, Reconstruction of R1 and R2 spherule in *Dscam*^*GoF*^ retina. **C and D**, Statistical analysis of R1 spherules between WT and *Dscam*^*GoF*^ retina show significant differences in all but invaginating bipolar cell dendrite volume. **E and F**, Statistical analysis of R2 spherules comparing WT and *Dscam*^*GoF*^ retina show insignificant differences in all but invaginating bipolar cell dendrite volume.

R1^GoF^ and R2^GoF^ spherules had a normal synaptic organization (Fig 6A and 6B). 3D reconstruction illustrated that R1^GoF^ terminals extended a long axon and displayed a spherical outline that was distinct from the R2^GoF^ spherule. The ribbon synapse site was consistently contacted by invagination of two bipolar cell dendrites and two horizontal cell axon tips (10/10 and 9/10). Statistical analysis indicated R1^GoF^ and R1^WT^ were significantly different in all volume measurements except bipolar cell dendritic invaginations (Fig 6C and 6D). R2^GoF^ spherules were very similar to the R2^WT^ spherule in volume, where cytoplasm, total spherule volume, invaginating horizontal cell axon tips and mitochondria size had no statistically significant differences (Fig 6C and 6D).

### Retracted rod spherules in *Dscam*^*GoF*^ retina are similar in size to R2 spherules but are internally disorganized

**Fig 7:**
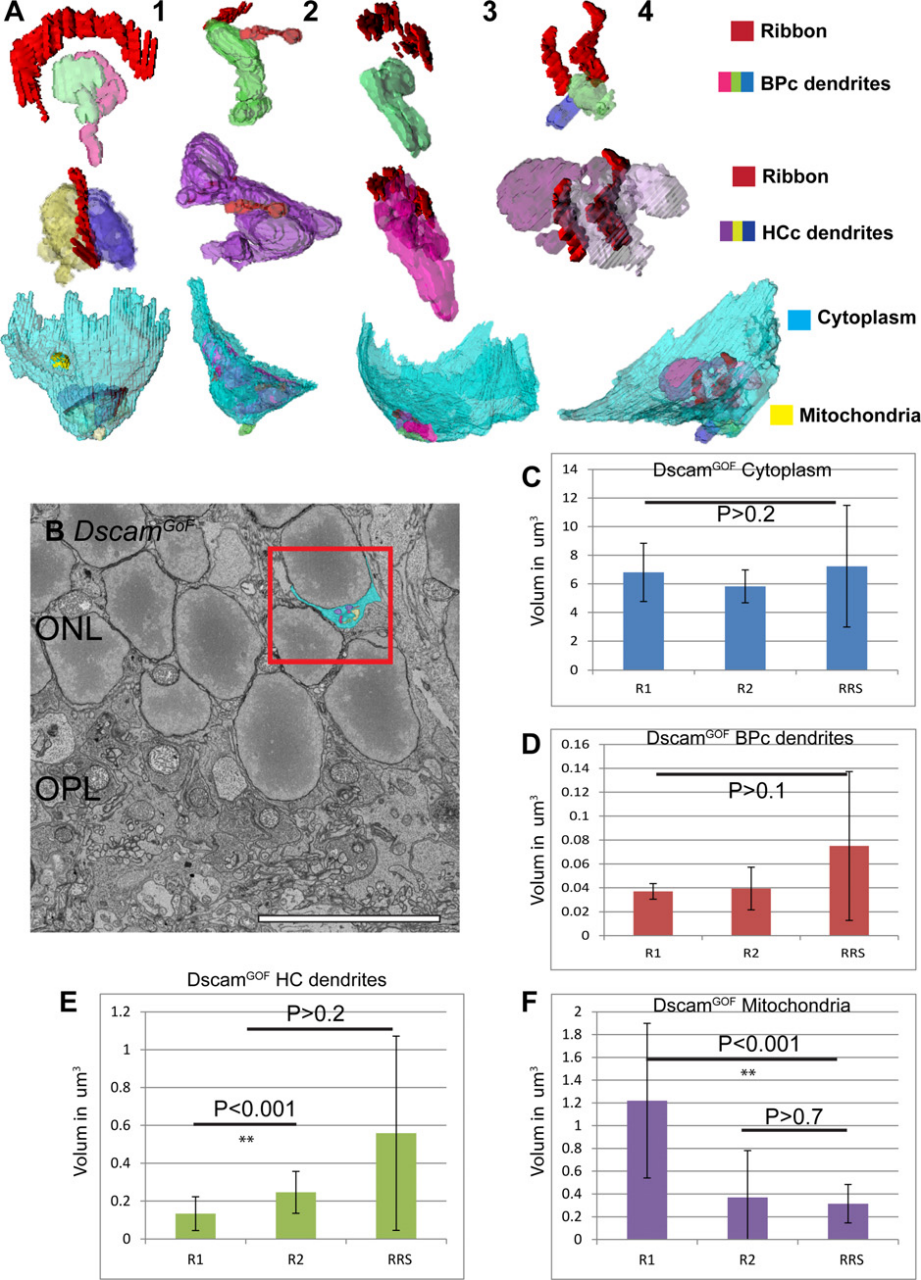
Retracted spherules in *Dscam*^*GoF*^ are similar to R2 spherules but with altered internal structures. **A**, Reconstruction of retracted rod spherules (RRS) in *Dscam*^*GoF*^ retina. Only one spherule shows normal morphology and the rest display different degrees of abnormal morphology in the size and orientation of the synaptic complex. **B**, Retracted spherules in *Dscam*^*GoF*^ retina were identified greater than one rod soma deep into the ONL. **C**, Statistical analysis of spherules types in *Dscam*^*GoF*^ retina revealed no difference in cytoplasm volume across spherule types. **D**, Statistical analysis of spherules types in *Dscam*^*GoF*^ retina revealed no difference in invaginating bipolar cell dendrite volume across rod types. **E**, Statistical analysis of spherule types in *Dscam*^*GoF*^ retina show significant differences between horizontal cell axon volume in R1 and R2 spherules but not compared to retracted spherules, likely due to the high variance in horizontal cell axon volume. **F**, Statistical analysis of mitochondrial volume across spherule types in *Dscam*^*GoF*^ retina show significant differences between R1 and other spherules (Bar in **B** is 10μm).

We further identified retracted rod spherules (RRS) that were at least 1–3 cells deep into the ONL and classified them as RRS^GoF^ (Fig 7B). We also identified gross morphology of the spherule and the ribbon. The outline of the displaced rod spherules consistently displayed a bowl shape that was similar to R2 spherules. However, 3D reconstruction indicated highly disorganized intraspherule structure. Out of four reconstructed displaced spherules, only one displayed a similar organization to R1 and R2 spherules with a crescent shaped ribbon contacting two invaginating bipolar cell dendrite tips and horizontal cell axon tips, and with a degree of invaginating symmetry. The rest of the spherules had fractured ribbons with sharp curvatures and extremely large or small invaginating neurites (Fig 7A 1–4).

We documented organizational differences in all reconstructed spherule types comparing WT and *Dscam*^*GoF*^. We found that in both R1 and R2 spherules, ribbon shape was preserved and there were occasionally one or three invaginations from bipolar and horizontal cells, but these occurrences were small compared to the overall sample sizes. Retracted spherules displayed abnormal ribbon morphology and inconsistent contact numbers from invaginating interneurons (Table 1).

**Table 1:** Abnormalities observed per spherule across genotypes and spherule types.

Spherule Type	Disorganized Ribbon	Atypical number of BPC invaginations	Atypical number of HC invaginations	Multiple mitochondria
R1^WT^	0/10	1/10	1/10	1/10
R2^WT^	0/10	1/10	0/10	3/10
R1^GoF^	0/10	0/10	1/10	3/10
R2^GoF^	0/10	2/10	1/10	2/10
RS^GoF^	3/4	3/4	3/4	1/4

### Data summary

**Fig 8:**
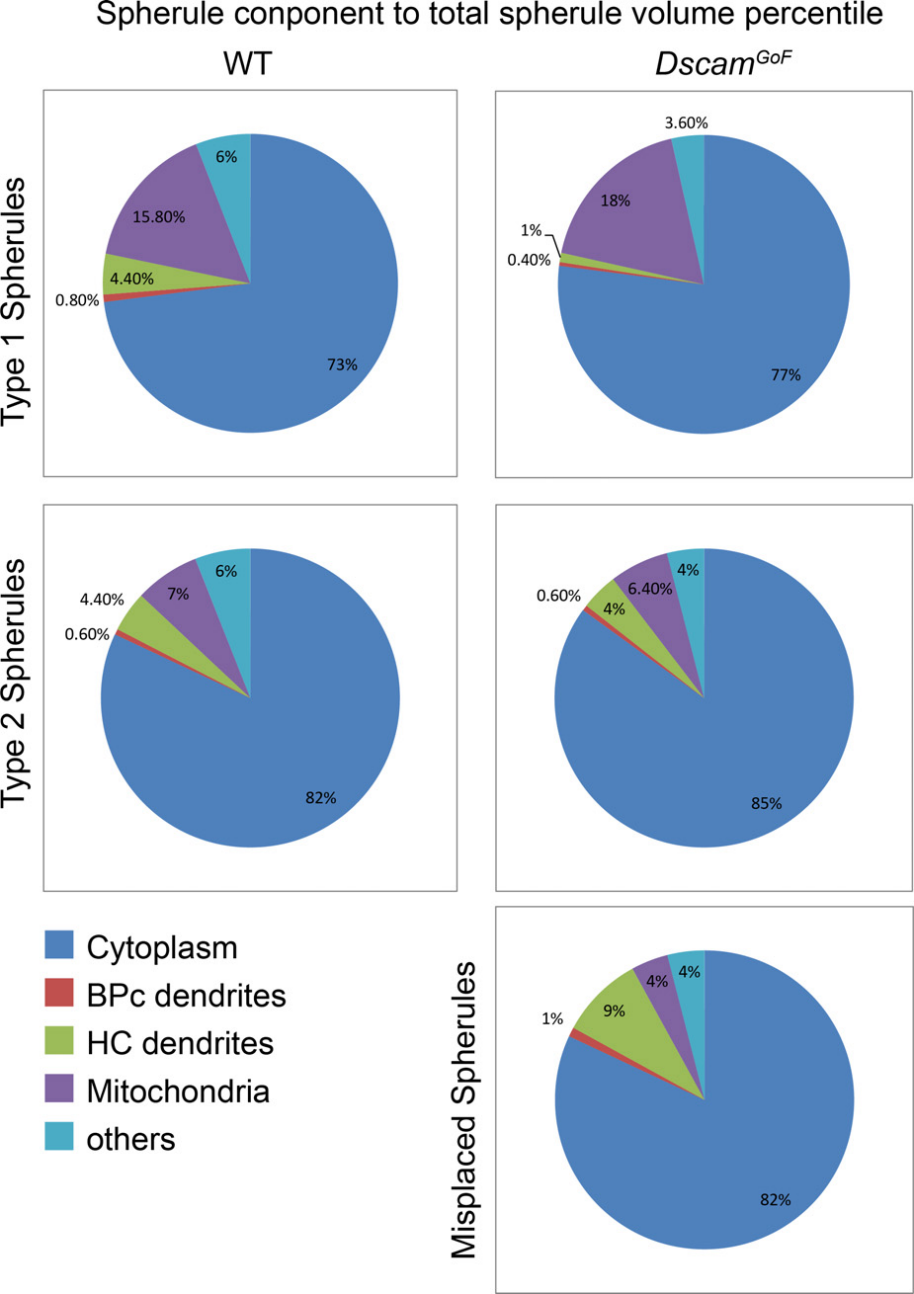
Percentile comparison illustrating spherule aspects. Pie graphs of the ratio of cellular components to the total spherule volume. We demonstrated that type 1 and type 2 spherules are closer in ratio perspective to each other in different genotypes than type 1 and type 2 spherules in the same genotype. Misplaced spherules are generally closer to type 2 spherules in ratio measurement.

The volumetric measurement gave evidence to support morphological differences between spherule types and validated abnormalities in the retracted spherules. Our analysis indicated R1 spherules were proportionally different from R2 spherules. R2 spherules in *Dscam*^*GoF*^ were not significantly different in respect to wild type R2 spherules, but the retracted spherules that they superficially resemble had notable disruption in the organization of their synaptic structures (Fig 8).

## Discussion

### Rod spherule diversity

In this study we characterized the two primary rod spherule morphologies in the mouse retina and compared them to the retracted rod spherule in a disease model. The *Mus musculus* retina has an axonless rod morphology, and we report that synaptic structures are preserved in this population. This structure superficially resembles the synaptic structure observed in multiple models of rod dysfunction. Despite the superficial resemblance between axonless rods and retracted spherules, we find significant changes in the synaptic structures of the retracted spherule that likely impinge upon their normal function.

Whether the different organization of rod spherules in the wild type retina have a developmental or functional role remains an open question. Our characterization of multiple rod types is consistent with the complexity of rod genesis and the formation of the ONL. It is known that this process can be influenced both intrinsically through genetic regulation and extrinsically through environmental and post-translational modification [36, 37]. ONL formation alone is largely dependent on the rod photoreceptors. For example, in the *Nrl* mutant mouse rod precursors develop into cones [38]. Without rods to maintain the tension between different outer retina layers, deformation of the retina is observed [39]. This demonstrates that rod cell fate does indeed have intrinsic clues to set its migrating path, define basal ONL boundary, and organize the outer retina architecture. The consistent coverage of R2 rods at the basal layer of ONL suggests ONL boundary formation as a potential avenue for future investigation. Future investigations of R1 and R2 spherules will also focus on the extent to which the respective rod types are contacted by OFF cone bipolar cells and the telodendria of cones and if the two spherule types are functionally different.

### Energy conservation and morphological efficiency in spherules

Misplaced rod spherules occur as the result of multiple pathologies. The large number of mutant retinas that display retraction of the rod spherule suggests that retraction may be an adaptation to stress that can be compensated for by eliminating the axon and adopting an R2 conformation. This could explain the common adaption to different types of stress observed in mouse mutants and in the aging retina. We had hypothesized that R2 spherules may be simpler structures for the rod to maintain, and in cases of stress, the rod adopts this morphology. This was not the case with respect to the mutant line examined in this study, as we found that synaptic organization was disrupted. We did find that mitochondria were significantly smaller in size in R2 spherules, consistent with these spherules being less metabolically demanding. The consistently large mitochondria in the type R1 spherule, 15% of the spherule volume, are likely necessary for local energy production because the synapse is away from cell soma. R2 spherules were adjacent to the cell soma and had smaller mitochondria consisting of only 6.67% of the spherule volume. This result suggests that it’s more efficient to produce energy locally for synaptic activity.

### *Dscam*^*GOF*^ rod spherules morphology could contribute to abnormal physiology

In a previous study we demonstrated *Dscam*^*GOF*^ retina had defects in OPL physiology in both light and dark adapted ERG recordings [27]. Here we find evidence that retracted rod spherules had internal disorganization that could contribute to this defect. However, it is likely that reduced bipolar cell number in this genetic background is also influencing physiology.

### Conclusion

In this study we reconstructed two types of rod spherule structure in wild type and *Dscam*^*GoF*^ transgenic mice. We found two major classes of rod spherule structure in the wild type retina, and these two structures are conserved in the *Dscam*^*GoF*^ retina. Retracted rod spherules in the *Dscam*^*GoF*^ retina superficially resembled R2 spherules but had abnormal organization of invaginating neurites.

## Materials and Methods

### Mouse and tissue processing

Wild type mice were maintained on a mixed C3H/HeJ and C57Bl/6J genetic background. *Dscam*^*GOF*^ mice were described previously [27]. Mice were anesthetized using tribromoethanol, which was administered by IP injection. Animals used for histochemistry in this study were perfused with PBS, and retinas were hemisected and fixed in 4% PFA for 30 minutes at room temperature. All procedures performed on mice used in this study were approved by the University of Idaho Animal Care and Use Committee.

### Antibodies and stains

Rabbit anti-calbindin (horizontal cells; Swant; CB38a; 1:1,000), Rabbit anti-cone arrestin (cones; Millipore; AB15282; 1:5,000), rabbit anti-PSD95 (synapses; Cell Signaling Technology; 1:1000), mouse anti-CTBP2 (synapses: BD Biosciences 1:1,000), peanut lectin (cone terminals: Millipore 1:2,000). Goat anti Bassoon (synaptic stains; Santa Cruz Biotechnology, Santa Cruz, CA; sc-18565; 1:400), and DAPI reagent (mixed into the second wash after incubation with secondary antibodies at a dilution of 1:50,000 of a 1 mg/ml stock). Secondary antibodies were acquired from Jackson Immuno Research and used at a concentration of 1:1000. Tissue was stained as previously described [40]. Briefly, antibodies were diluted in a blocking solution of 0.1% triton (sections) or 0.4% triton (whole retinas) supplemented with 7% normal donkey serum in PBS. Sections were blocked for 20 minutes in blocking solution and then incubated in primary antibody overnight at 4° C or at room temperature for one hour. Whole retinas were blocked for two hours in blocking solution incubated at 4° C for four days. Sections were washed 3 x for five minutes in PBS, while the whole retina washes were carried out for one hour. Tissue was incubated in secondary antibody diluted in blocking solution, as performed for primary antibodies, washed and mounted in 80% glycerol.

### EM and confocal imaging

Block face EM images were acquired through Renovo neural EM services (Cleveland, Ohio) using a Zeiss Sigma VP scanning electron microscope. Tissues were prepared according to company directions. Briefly, mice were perfused with cacodylate buffer and retina pieces were collected from the midway point between the optic nerve and periphery. Four electron micrograph volumes were collected each from two wild type and *Dscam*^*GoF*^ mice. Retinas were fixed for four days in cacodylate buffer before processing. Sections were imaged at a resolution of 7 nm with 60 nm steps in between slices.

Confocal microscopy was performed using an Olympus Fluoview or Olympus DSU spinning disk microscope. Any modification to images were performed across the entire image, in accordance with PLoS journal standards.

### Statistical analysis

Two sample comparisons in this study were done by Student *t*-tests, multiple sample comparison was performed with one way Anova, P values and F values are given in each test performed.

### Software used in this study

The TrakEM2 software used in this study enabled the analysis of photoreceptor spherule morphology through 3D reconstruction and volume assemblage [41, 42]. The TrakEM2 0.9a User Manual was followed for all procedures in the study. The electron microscopy image sets were first imported into the TrakEM2 canvas from Fiji; the stack slices were then aligned in order to obtain accurate volumetric measurements and reconstructions. We used affine invariant matching as the expected transformation for alignment.

Once aligned, the process of tracing rod spherules was performed. Different area lists were created for independent cellular structures; these structures were then individually traced for reconstruction and volumetric purposes. Once the cellular structures were adequately traced in the TrakEM2 canvas, 3D models were resampled at 5:1 pixel ration to reduce rendering time. These 3D reconstructions included single and multiple organelles, and entire photoreceptor spherules. 3D reconstructions then used “smooth mesh” functions to realistically represent the membrane morphologies.

## Supporting Information

S1 Fig **R2 rods in *Mus*. A** and **B**, Additional 3D reconstruction of surface rods adjacent to OPL in wild type mouse retina. **C** and **D**, EM of the wild type mouse retina illustrated a consistent percent of R2 rods in different animals. Yellow highlight refers to R2 rod cytoplasm and blue highlight refers to R1 rod cytoplasm. **E**, 95% (92 out of 97 cells counted) of R2 spherules have an immediate ribbon close to the cell soma. Scale bars are 10 μm in **A** and **C**.
(TIF)

S1 Movie Rod Ribbon and Bipolar Cell Dendrite Tips. Ribbon structure (top) is colored purple. Paired bipolar cell dendrites (bottom) are colored plum and lilac.
(AVI)

S2 Movie Rod Ribbon and Horizontal Cell Axon Tips. Ribbon structure (purple) is contacted by horizontal cell axon tips (yellow and buff).
(AVI)

S3 Movie R1 Spherule Reconstruction. Axon (red) connects to rod soma (not shown) and spherule (cyan). Mitochondria (orange) is proximal to the axon compared to invaginating neurites and ribbon.
(AVI)

S4 Movie R2 Spherule Reconstruction. Spherule hugs rod nucleus (not shown). Mitochondria are orange. Ribbon (purple) and neurites (yellow) are distal from the cell soma.
(AVI)

S5 Movie Apical Layer of Rod Soma and Associated Ribbons. Rod Soma (cyan) and ribbons (red).
(AVI)

S6 Movie Reconstruction of 2 R2 and 1 R1 Spherules for Comparison. R1 spherules are attached to axons (red).
(AVI)

S7 Movie Reconstructed R1 and R2 spherules in the *Dscam*^*GOF*^ retina. R1 and R2 type spherules for comparison.
(AVI)
