# Supplementary figures and images for: Morphological Diversity of the Rod Spherule: A Study of Serially Reconstructed Electron Micrographs
Source: PLoS One. 2016 Mar 1;11(3):e0150024. doi: 10.1371/journal.pone.0150024 (PMC4773090; doi:10.1371/journal.pone.0150024)

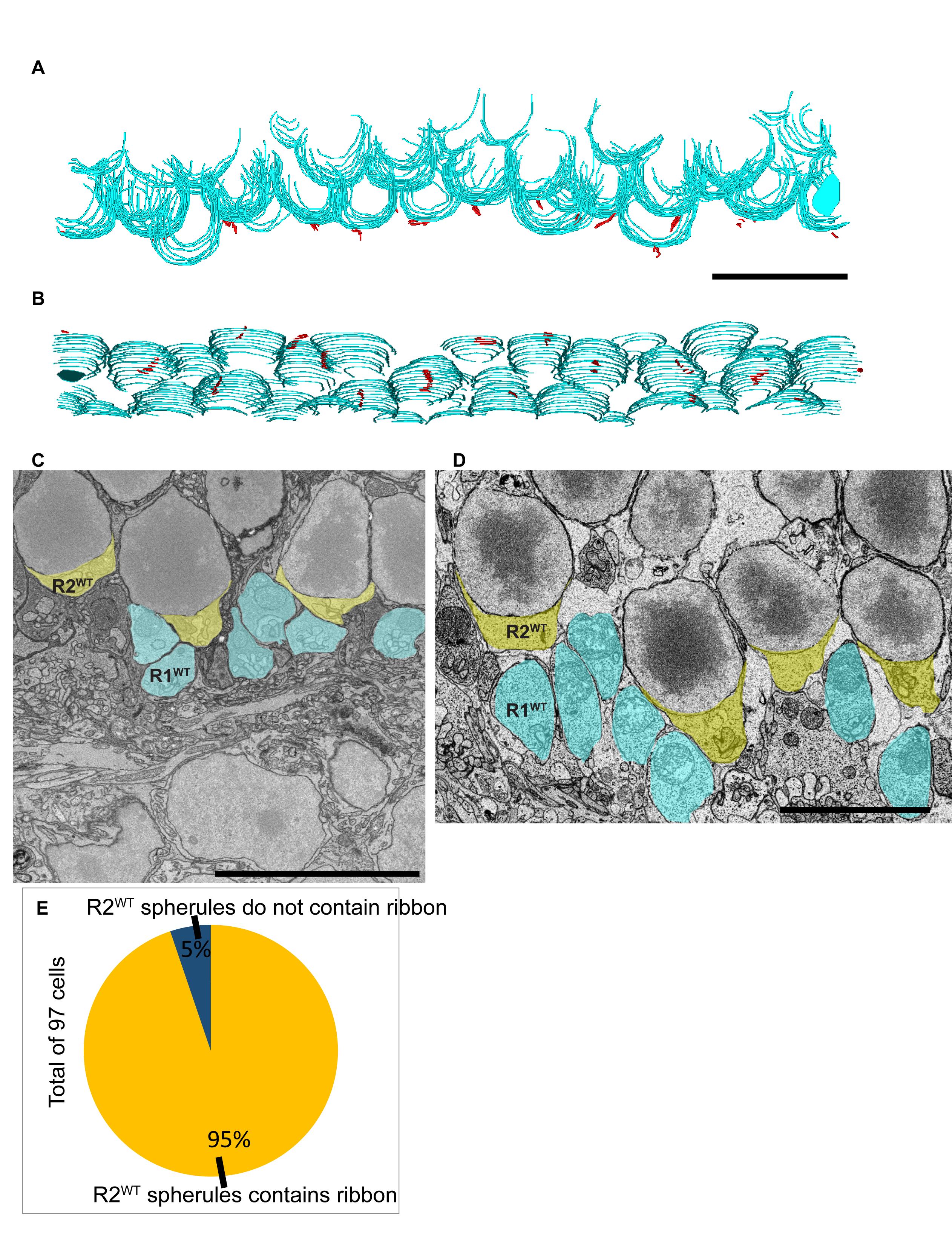

Supplement: S1 Fig — R2 rods in Mus. A and B, Additional 3D reconstruction of surface rods adjacent to OPL in wild type mouse retina. C and D, EM of the wild type mouse retina illustrated a consistent percent of R2 rods in different animals. Yellow highlight refers to R2 rod cytoplasm and blue highlight refers to R1 rod cytoplasm. E, 95% (92 out of 97 cells counted) of R2 spherules have an immediate ribbon close to the cell soma. Scale bars are 10 μm in A and C. (TIF) [file pone.0150024.s001.tif]
